# Beta-blocker use and breast cancer outcomes: a meta-analysis

**DOI:** 10.1007/s10549-024-07263-4

**Published:** 2024-06-05

**Authors:** Oliver William Scott, Sandar TinTin, Alana Cavadino, J. Mark Elwood

**Affiliations:** Department of Epidemiology and Biostatistics, School of Population Health, University of Auckland, Building 507, 85 Park Road, Grafton, Auckland, 1023 New Zealand

**Keywords:** Breast cancer, Mortality, Beta blockers, Pharmacoepidemiology, Meta analysis

## Abstract

**Purpose:**

Beta blockers (BBs) are commonly used cardiovascular medications, and their association with breast cancer outcomes has been examined in several previous observational studies and meta-analyses. In this study, an updated meta-analysis was undertaken to ascertain the association between BBs and both breast cancer death (BCD) and breast cancer recurrence (BCR).

**Methods:**

Articles were sourced from various databases up until the 14th of August 2023. Effect estimates were pooled using the random effects model, and the Higgins I^2^ statistic was computed to ascertain heterogeneity. Subgroup analyses were conducted by the potential for immortal time bias (ITB), the exposure period (prediagnosis vs postdiagnosis), and type of BB (selective vs non-selective). Publication bias was assessed using funnel plots and Egger’s regression tests.

**Results:**

Twenty-four studies were included. Pooled results showed that there was no statistically significant association between BB use and both BCD (19 studies, hazard ratio = 0.90, 95% CI 0.78–1.04) and BCR (16 studies, HR = 0.87, 95% CI 0.71–1.08). After removing studies with ITB, the associations were attenuated towards the null. There was no effect modification for either outcome when stratifying by the exposure period or type of BB. There was clear evidence of publication bias for both outcomes.

**Conclusion:**

In this meta-analysis, we found no evidence of an association between BB use and both BCD and BCR. Removing studies with ITB attenuated the associations towards the null, but there was no effect modification by the exposure period or type of BB.

**Supplementary Information:**

The online version contains supplementary material available at 10.1007/s10549-024-07263-4.

## Introduction

Breast cancer is the most common cancer in women and the leading cause of female cancer mortality worldwide [1]. Comorbidities are common in patients with breast cancer [2], and there is a high and increasing prevalence of risk factors for both breast cancer and ischemic heart disease among Western women [3–5]. As such, many patients with breast cancer use prescribed medications for cardiovascular conditions. Ascertaining the association between commonly used cardiovascular medications and breast cancer outcomes is therefore warranted. Beta blockers (BBs), principally indicated for angina, arrhythmias, heart failure, hypertension, and myocardial infarction [6, 7], are commonly used cardiovascular medications that have been used in Western medicine for decades [8–10].

Human breast cancer cells have beta-adrenergic receptors [11]. Responses induced by beta-adrenergic signalling include upregulated expression of metastasis-associated genes involved in inflammation, angiogenesis, and tissue invasion, and downregulated expression of genes facilitating anti-tumour immune responses [12]. Beta-adrenergic receptors mediate the catecholamine hormones produced in the stress response, which in turn produce the aforementioned responses [13]. These hormones can be blocked by BBs, resulting in a potential protective effect through interference with tumour cell proliferation and migration, as well as tumoural angiogenesis [14, 15]. On the basis of this evidence, a number of observational studies have been carried out, and several meta-analyses have been published to summarise these studies [16–22]. Published between 2015 and 2023 and covering studies published up until the 1st of January 2023, most of these meta-analyses have shown no statistically significant association between the use of BBs and the prognosis of breast cancer.

In the meta-analyses published to date, some of the more recent observational studies have not been included [23, 24]. Further, there have not been many meta-analyses that have examined potentially important effect modifiers of the association such as the presence of immortal time bias (ITB), the type of BB (selective vs non-selective), or the exposure period in which BBs were used (prediagnosis vs postdiagnosis). ITB, for example, is a bias in which a spurious survival advantage is invariably conferred to the user group in studies that count user time in a period where events could not occur by design [25]. Further, several preclinical studies have suggested that non-selective BBs may have a higher efficacy in inhibiting pathways involved in breast cancer progression and metastasis [15, 26]. It has also been suggested that BBs taken prior to breast cancer diagnosis (and around the time of diagnosis) may be more efficacious in preventing breast cancer progression/metastases than BBs taken after diagnosis [15, 27–33]. As such, the objective of the current paper was to perform an updated meta-analysis examining the association between BBs and both breast cancer death (BCD) and breast cancer recurrence (BCR), and also examine whether the associations differ across potentially important subgroups.

## Methods

### Literature search/data sources

PubMed, Embase, Medline, Web of Science, Google Scholar, and the Cochrane Library databases were searched using the keywords [beta, beta blocker, beta antagonist, metoprolol, propranolol, carvedilol, labetalol, celiprolol, acebutolol, bisoprolol, breast cancer, breast carcinoma, cancer, carcinoma, recurrence, metastasis, outcome, death, prognosis, survival, mortality, proliferation] in combinations of ‘AND’ or ‘OR’. There was no restriction placed on the earliest date of publication, but articles were only selected for review if they analysed human breast cancer patients and were written in English. Further articles were sourced from scanning the reference lists of individual observational studies and meta-analyses found using the aforementioned search criteria. Literature was sourced up until the 14th of August 2023.

### Study selection/eligibility criteria

Studies were eligible for inclusion if they met the following criteria: (1) Included women with breast cancer as the cohort of interest; (2) Analysed data from cohort studies, case–control studies, or randomised controlled trials (including retrospective analyses of already published RCTs); (3) Assessed BB use as the exposure of interest (with no minimum dose requirement); (4) Reported BCD or BCR (or both) as the outcome(s) of interest; (5) Reported risk estimates and 95% confidence intervals for the associations of interest; and (6) Were published as full length articles. We did not consider systematic reviews, laboratory experiments, case reports, ecological studies, conference abstracts, or editorials for inclusion in this meta-analysis.

### Data extraction

The titles and abstracts of each individual study were firstly examined to assess their eligibility, and relevant information from each study was extracted. Information extracted from each study included the first author and year of study, country of study, the number of women under study, study design, patient characteristics (such as cancer stage and age), exposure definition (pre or postdiagnosis), the source of exposure data, median or mean follow-up time, the potential for ITB (studies were judged to be susceptible to ITB when they used a ‘time fixed’ postdiagnosis definition of medication use), covariates adjusted for, the types of BBs used by women (e.g., all BBs, selective BBs, non-selective BBs, or a combination), and the reference group used (e.g. BB users vs all BB non-users or BB users vs a different comparison group). When studies used a stepwise approach for covariate adjustment, the fully adjusted HR was taken.

### Statistical analysis

The main analysis assessed the association between BB use at any time and breast cancer outcomes, pooling the data from all included studies. When studies reported risk estimates for BBs used both before and after breast cancer diagnosis for the same cohort, only the risk estimates for postdiagnosis use were taken (to avoid sample overlap), as it can be considered that postdiagnosis use of medications over the course of breast cancer therapy is the more clinically relevant exposure period. Risk estimates and 95% confidence intervals were transformed onto the log scale, as recommended [34]. To pool risk estimates into a summary estimate, the inverse variance method with random effects model was used. We used a random effects model because it seems plausible that the effect of BBs could vary from study to study due to factors other than sampling variability [35]. In the paper by Lorona and others [36], the HR for the association between BB use and both BCD and BCR was initially stratified by breast cancer molecular subtype (luminal, triple negative, and HER2 positive), and no overall HR was reported. These HRs were firstly pooled using a random effects model to derive an overall summary estimate for the association among all types of breast cancer, and the resulting pooled HR was then included in this meta-analysis. A similar method was used to pool results for the association between prediagnosis BB use and BCR in the paper by Sorensen and colleagues [37], in which the HR for prediagnosis use was initially stratified by the number of prescribed tablets. The Higgins I^2^ statistic was computed for each pooled estimate to ascertain the amount of heterogeneity present between studies, and an I^2^ statistic of > 50% indicated that there was a significant amount of heterogeneity [38].

To explore reasons for heterogeneity between studies and to assess the impact of different potential modifying variables on the associations of interest, subgroup analyses [39] were carried out by the potential for ITB (yes vs no), the exposure period in which BBs were used (prediagnosis vs postdiagnosis), and the type of BB used (selective vs non-selective). Because of the bias inherent in studies judged to be susceptible to ITB [25], the Subgroup analyses by exposure period and type of BB used were restricted to studies judged not to be susceptible to ITB. Studies were excluded in the subgroup analysis by exposure period if they assessed BB use both before and after diagnosis in the same analysis. To formally test for differences between subgroups, a random effects meta regression was used [40]. Sensitivity analyses were conducted to iteratively assess the impact of excluding each individual study on the overall summary estimate [41]. Lastly, funnel plots [42] were generated, and Egger’s regression tests [43] were performed to assess the presence of any potential publication bias. All reported p-values are two sided and were considered statistically significant if *p* < 0.05. All analyses were conducted in STATA 17.0 (StataCorp, College Station, TX).

## Results

### Literature search

**Fig. 1:**
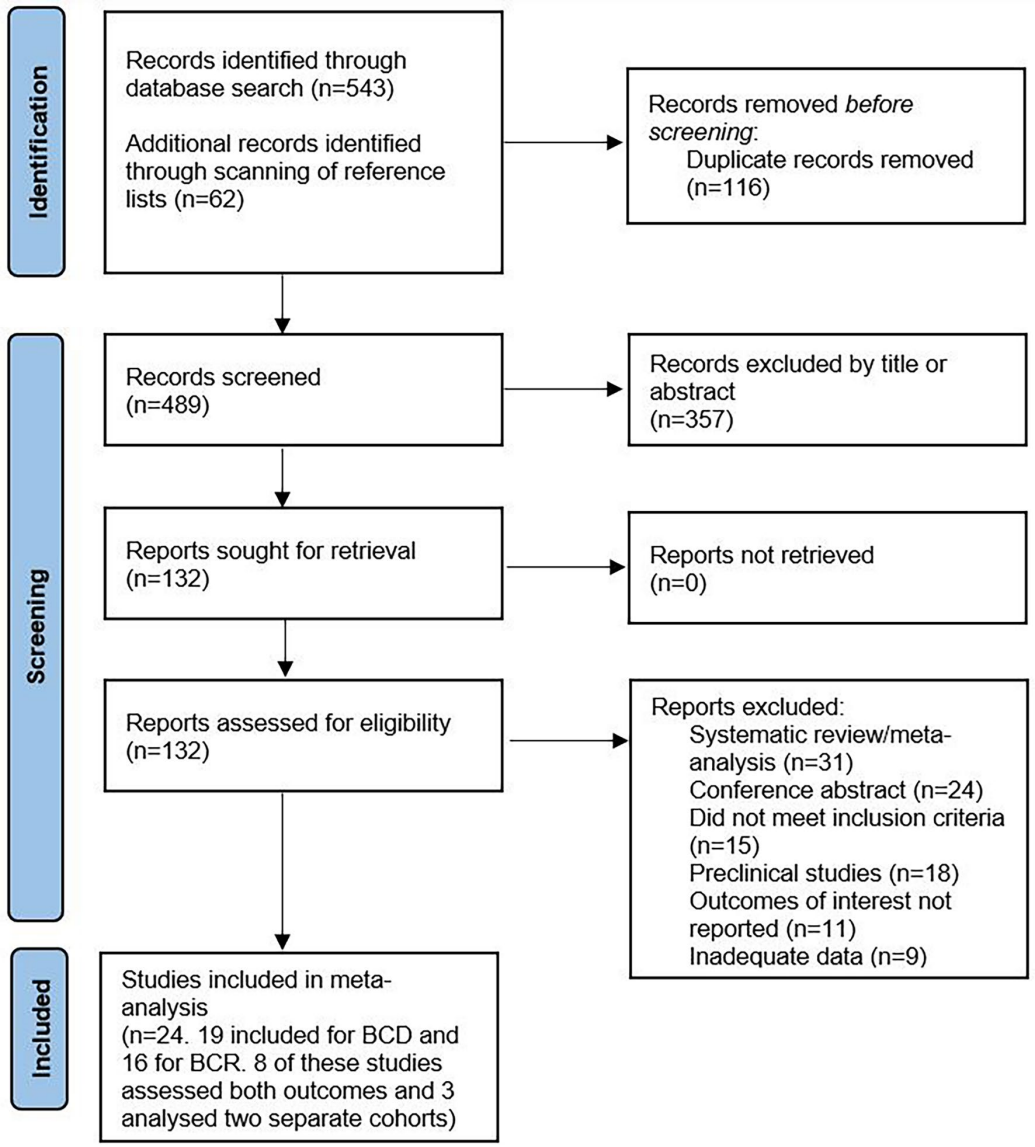
PRISMA chart of study selection

A total of 489 studies (after removing duplicates) were retrieved from the selected databases during the literature search and included for initial screening (Fig. 1). 132 reports were assessed in full, and after making various exclusions, 24 studies were deemed to meet the inclusion criteria and were included in this meta-analysis [23, 24, 36, 37, 44–63]. The detailed characteristics of these 24 studies are shown in Table 1.

**Table 1:** Characteristics of the included studies

First author and year of study	Country of study	Number under study	Study design	Patient characteristics	Exposure definition	Source of exposure data	Follow up time (mean or median, in years)	Potential for immortal time bias	Covariates adjusted for	Types of BB used by women in study	Reference group used	Study period (first date of BC dx until end of follow up where stated, otherwise dx dates of BC patients included)	Outcome(s) reported (relevant to this meta-analysis)	Effect estimates
Chang (2023) [23]	Italy and Norway (separate cohorts)	1135 and 907 in Italy and Norway respectively	Retrospective cohort	Women with all stages of BC (but only TNBC). Median ages of 63 and 60 years for BB users and nonusers in the Italian cohort respectively. Median ages of 66 and 58 years for BB users and nonusers in the Norwegian cohort respectively	BB use in the 3-month period prior to BC diagnosis	Combination of prescription records and medical records	Not reported	No	Italy-age, tumour dimension, lymph node involvement, statin use, aspirin use, other antihypertensive use, Ki-67 score, and peritumoral vascular invasion Norway-age, tumour dimension, lymph node involvement, statin use, aspirin use, antihypertensive use, and grade	All BBs	All non-users of BBs	1997–2021 (Italy), 2004–2018 (Norway)	BCD and BCR	0.59 (0.34–1.02) and 0.46 (0.22–0.95) for BCD for the Italian and Norwegian cohorts respectively. 0.55 (0.32–0.95) for BCR in the Italian cohort (BCR not reported for the Norwegian cohort)
Hsieh (2023) [24]	Taiwan	221	Retrospective cohort	Women with advanced stage BC. Mean age of 56.1 years	BB use after diagnosis of BC	Medical records	3.3	No	Age, ECOG performance status, number of metastases, and brain metastases	All BBs	All non-users of BBs	2012–2022	BCR	2.21 (1.56–3.12)
Lofling (2022) [57]	Norway	30,060	Retrospective cohort	Women with all stages of BC. Median age of 62, 67, and 69 years for nonusers, users of nonselective BBs, and users of selective BBs respectively	BB use in the 3-month period prior to BC diagnosis	Prescription records	5.1	No	Age, subtype, histology, year of diagnosis, education, marital status, number of children, country of origin, use of ACEI, use of ARB, use of CCB, use of diuretics, use of aspirin, use of NSAIDs, use of statins, and use of antidiabetics	All BBs, selective and non-selective in sensitivity analysis	All non-users of BBs, different comparison group in sensitivity analysis	2004–2018	BCD	1.07 (0.97–1.19)
Scott (2022) [58]	New Zealand	14,976	Retrospective cohort	Women with all stages of BC. Mean age of 58.3 years	BB use both before (in the 1-year period prior to diagnosis) and after diagnosis of BC	Prescription records	4.51	No	Age, date of diagnosis, ethnic group, deprivation, urban/rural status, public/private status, register, stage, grade, mode of detection, lymphovascular invasion, receptor status, statin use, NSAID and aspirin use, ACEI use, ARB use, diuretic use, cardiac conditions, diabetes, stroke, COPD, and peripheral vascular disease	All BBs, selective and non-selective in sensitivity analysis	All non-users of BBs, different comparison group in sensitivity analysis	2007–2017	BCD and BCR	1.05 (0.88–1.24) and 0.98 (0.81–1.18) for BCD and BCR respectively (prediagnostic) 1.11 (0.95–1.29) and 1.06 (0.90–1.25) for BCD and BCR respectively (postdiagnostic)
Gillis (2021) [56]	Norway	4014	Retrospective cohort	Women with stage I–III BC. Mean age of 68 years	BB use prior to BC diagnosis (counted as a user if the prescription covered the date of dx)	Prescription records	5.5	No	Age, education, region, stage, diabetic drug use, cardiac therapy drug use, and lipid-modifying agent use	Carvedilol (non-selective)	All non-users of BBs who used another antihypertensive	2007–2017	BCD	0.41 (0.15–1.09)
Lorona (2021) [36]	USA	4557	Prospective cohort	Women with all stages of BC. Median age of 52.0 years	BB use in the 6-month period prior to one month before BC diagnosis	Medical records	Not reported	No	Age, stage, local treatment, chemotherapy, race, BMI, use of diabetes medications, use of lipid lowering medications, hormone treatment, and trastuzumab treatment	All BBs	All non-users of antihypertensives	2004–2015	BCD and BCR	1.22 (0.87–1.73) and 1.30 (0.85–1.98) for BCD and BCR respectively
Santala (2020) [55]	Finland	73,170	Retrospective cohort	Women with all stages of BC. Median age of 66 years	BB use both before (any time) and after BC diagnosis	Prescription records	6.2	No	Age, tumour extent, primary treatment, obesity, participation in national screening program, use of hormone-receptor antagonists, and Charlson comorbidity score	All BBs, selective and non-selective in sensitivity analysis	All non-users of antihypertensives, all non-users of BBs who used another antihypertensive in sensitivity analysis	1995–2013	BCD	0.95 (0.91–1.00) and 0.92 (0.88–0.97) for prediagnostic and postdiagnostic use respectively
Cui (2019) [54]	China	633	Prospective cohort	Women with all stages of BC. Mean age not reported	BB use after diagnosis of BC	Combination of prescription records and self-report	6.3	No	Age, education, income, BMI, alcohol intake, smoking, year of diagnosis, stage, treatment, ARB use, ACEI use, CCB use, diuretic use, and Charlson comorbidity score	All BBs	All non-users of antihypertensives	1996–2014	BCD	1.26 (0.68–2.34)
Musselman (2018) [53]	Canada	4876	Retrospective cohort	Women with all stages of BC. Mean age of 76.2 years	BB use in the 90-day period prior to BC surgery (i.e., predominantly after diagnosis)	Prescription records	4.8	No	Age, socioeconomic status, and Charlson comorbidity score	All BBs, selective and non-selective in sensitivity analysis	All non-users of BBs who used another antihypertensive	2002–2010	BCD	1.03 (0.83–1.29)
Chen (2017) [52]	USA	14,766	Retrospective cohort	Women with stage I–II BC. Mean age not reported	BB use after diagnosis of BC	Prescription records	3.0	No	Age, stage, hormone receptor status, cancer treatments, comorbidities, and concurrent medications	All BBs, selective and non-selective in sensitivity analysis	All non-users of BBs, different comparison group in sensitivity analysis	2007–2012	BCD and BCR	1.41 (1.07–1.84) and 1.08 (0.91–1.27) for BCD and BCR respectively
Spera (2017) [63]	Canada	1144 and 3298 in the TRIO-012 and BCIRG-005 trials respectively	Retrospective analysis of a clinical trial	Women with advanced stage BC and early-stage BC in the TRIO-012 and BCIRG-005 trials respectively. Mean ages not reported	BB use both prior and after randomisation (both after diagnosis)	Prescription records	2.1 and 2.3 in the TRIO-012 and BCIRG-005 trials respectively	Yes	Age, treatments, and national region	All BBs	All non-users of BBs	NA	BCR	0.81 (0.66–0.99) and 0.75 (0.54–1.02) in the TRIO-012 and BCIRG-005 trials respectively
Cardwell (2016) [51]	Europe (pooled data)	55,252	Retrospective cohort	Women with all stages of BC. Mean age not reported	BB use both before (in the 1-year period prior to diagnosis) and after diagnosis of BC	Combination of prescription records and medical records	4–6 depending on location	No	Age, year of diagnosis, stage, grade, surgery, radiotherapy, chemotherapy, tamoxifen use, aromatase inhibitor use, aspirin use, statin use, HRT use, and comorbidities	Propranolol and other non-selective BBs	All non-users of propranolol	1998–2015	BCD	1.03 (0.86–1.22) and 0.94 (0.77–1.16) for prediagnostic and postdiagnostic use respectively
Choy (2016) [62]	USA	1029	Retrospective cohort	Women with stage II–III BC. Median age of 53 years	BB use in the 45-day period prior to BC surgery (i.e., predominantly after diagnosis)	Combination of prescription records and medical records	Not reported	No	Not reported	All BBs (none that were indicated for glaucoma)	All non-users of BBs	2000–2010	BCR	0.51 (0.23–0.97) for stage II patients. Not reported for stage III patients
Boudreau (2014) [60]	USA	4216	Retrospective cohort	Women with stage I–II BC. Mean age of 63.0 years	BB use after diagnosis of BC	Prescription records	6.3	No	Age, BMI, stage, hormone receptor status, menopausal status, Charlson comorbidity score, diabetes, cancer treatments, and concurrent medications	All BBs	All non-users of BBs	1990–2008	BCR	1.29 (1.01–1.64)
Sakellakis (2014) [61]	Greece	610	Retrospective cohort	Women with stage I–III BC. Mean age of 60.8 years	BB use after diagnosis of BC	Medical records	3.8	No	Age, stage, and hormone receptor status	All BBs	All non-users of BBs	1983–2013	BCR	0.85 (0.54–1.34)
Botteri (2013) [47]	Italy	800	Retrospective cohort	Postmenopausal women with stage I–III TNBC. Mean age of 59.1 years	BB use at the time of breast cancer diagnosis (no timeframe used)	Medical records	6.0	No	Age, stage, cancer treatment, and concurrent medications	All BBs	All non-users of BBs	1997–2008	BCD and BCR	0.42 (0.18–0.97) and 0.52 (0.28–0.97) for BCD and BCR respectively
Cardwell (2013) [48]	England	7132	Nested case control	Women with all stages of BC. Mean age not reported	BB use both before (in the 1-year period prior to diagnosis) and after diagnosis of BC	Prescription records	3.9	No	Age, stage, tamoxifen use, cancer treatments, comorbidities, and concurrent medications	All BBs, non-selective in sensitivity analysis	All non-users of BBs, different comparison group in sensitivity analysis	1998–2007	BCD	0.97 (0.83–1.14) and 1.20 (0.92–1.57) for prediagnostic and postdiagnostic use respectively
Chae (2013) [49]	USA	,449	Prospective cohort	Women with all stages of BC. Mean age of 50.3 years	BB use after diagnosis of BC	Combination of prescription records and medical records	4.6	Yes	Age, race, BMI, stage, grade, and concurrent medications	All BBs	All non-users of BBs	1995–2007	BCD and BCR	0.51 (0.27–0.96) and 0.51 (0.29–0.87) for BCD and BCR respectively
Holmes (2013) [50]	USA	4661	Prospective cohort	Women with stage I–III BC. Mean age of 63.3 years	BB use after diagnosis of BC	Self-report	10.5	No	Age, stage, BMI, menopausal status, oral contraceptive use, cancer treatment, and concurrent medications	All BBs	All non-users of BBs	1988–2008	BCD	0.83 (0.60–1.16)
Sørensen (2013) [37]	Denmark	18,733	Prospective cohort	Women with stage I–III BC. Mean age of 60.2 years	BB use both before (in the 3-year period prior to diagnosis) and after diagnosis of BC	Prescription records	6.8	No	Age, menopausal status, stage, grade, hormone receptor status, cancer treatment, and concurrent medications	All BBs, selective and non-selective in sensitivity analysis	All non-users of BBs	1996–2010	BCR	1.10 (0.92–1.32) and 1.30 (1.10–1.50) for prediagnostic and postdiagnostic use respectively
Barron (2011) [45]	Ireland	1575 (atenolol), 210 (propranolol)	Retrospective cohort	Women with all stages of BC. Mean ages of 71.0 and 69.0 years for the atenolol and propranolol cohorts respectively	Use of the respective medication in the 1-year period prior to BC diagnosis	Prescription records	2.7 and 3.5 for the atenolol and pro propranolol cohorts respectively	No	Age, stage, grade, and comorbidities	Propranolol and atenolol	All non-users of BBs	2001–2007	BCD	0.19 (0.06–0.60) for propranolol and 1.08 (0.84–1.40) for atenolol
Ganz (2011) [46]	USA	1779	Retrospective cohort	Women with stage I–IIIA BC. Mean age not reported	BB use both before and after diagnosis of BC (in the same analysis)	Prescription records	8.2	No	Age, race, stage, BMI, cancer treatment, hormone receptor status, tamoxifen use, hypertension, and diabetes	All BBs	All non-users of BBs who used another antihypertensive	1997–2010	BCD and BCR	0.76 (0.44–1.33) and 0.86 (0.57–1.32) for BCD and BCR respectively
Melhem-Bertrandt (2011) [59]	USA	1413	Retrospective cohort	Women with stage I–III BC. Mean age of 49.4 years	BB use after diagnosis of BC	Combination of prescription records, medical records, and self-report	5.1	Yes	Age, race, stage, grade, hormone receptor status, BMI, comorbidities, and concurrent medications	All BBs	All non-users of BBs	1995–2007	BCR	0.52 (0.31–0.88)
Powe (2010) [44]	England	466	Prospective cohort	Women with stage I-II BC. Mean age of 57.0 years	BB use after diagnosis of BC	Medical records	10.0	Yes	Age, stage, grade, and tumour size	All BBs	All non-users of BBs who used another antihypertensive	1987–1994	BCD and BCR	0.29 (0.12–0.72) and 0.43 (0.20–0.93) for BCD and BCR respectively

### Study characteristics

Overall, this meta-analysis included 24 studies [23, 24, 36, 37, 44–63] with a combined total of 253,082 women with breast cancer. Nineteen of these studies assessed BCD as an outcome, while sixteen assessed BCR as an outcome (eight of these studies assessed both outcomes). There were three studies that included two different cohorts/datasets, these being the studies by Spera et al. [63] (in which they retrospectively analysed two different clinical trials), Barron et al. [45] (in which they analysed propranolol and atenolol in two separate cohorts), and Chang et al. [23] (in which cohorts from both Italy and Norway were analysed). As such, there were 27 unique datasets available for this meta-analysis. Eleven of the 24 studies were conducted in Europe (including the UK), ten in North America, and three were from the Asia/Oceania region. Sixteen were designed as retrospective cohort studies, six were prospective cohort studies, one was a nested case control study, and one retrospectively analysed two clinical trials. Almost all studies (except one [62]) adjusted for covariates, and every study that adjusted for confounders adjusted for age at the very least. In general, a range of confounders were usually adjusted for, including demographic variables, breast cancer clinical variables, comorbidities, and concomitant medication usage. For example, potentially important confounders such as cancer stage, comorbidities, and concomitant medication use were adjusted for in 17/24, 11/24, and 15/24 studies respectively. The mean or median follow-up time across the 24 studies ranged from 2.1 to 10.5 years, with ten studies reporting a mean/median follow-up time of 0–5 years, nine reporting a mean/median follow-up time of 5–10 years, and two reporting a mean/median follow-up time of 10 + years. Follow-up time was not reported in three of the studies. Seventeen studies analysed BB use after the diagnosis of breast cancer, six considered prediagnosis BB use as the exposure of interest, and one considered BB use both before and after diagnosis in the same analysis. Twelve studies ascertained medication use through prescription records, five through medical records, one through self-reported patient data, and six through a combination of data sources. Twenty studies were judged not to be susceptible to ITB, while four were. Finally, there were eleven studies that stratified BB use by type (selective vs non-selective), while thirteen did not.

### Association between BB use and breast cancer related deaths

**Fig. 2:**
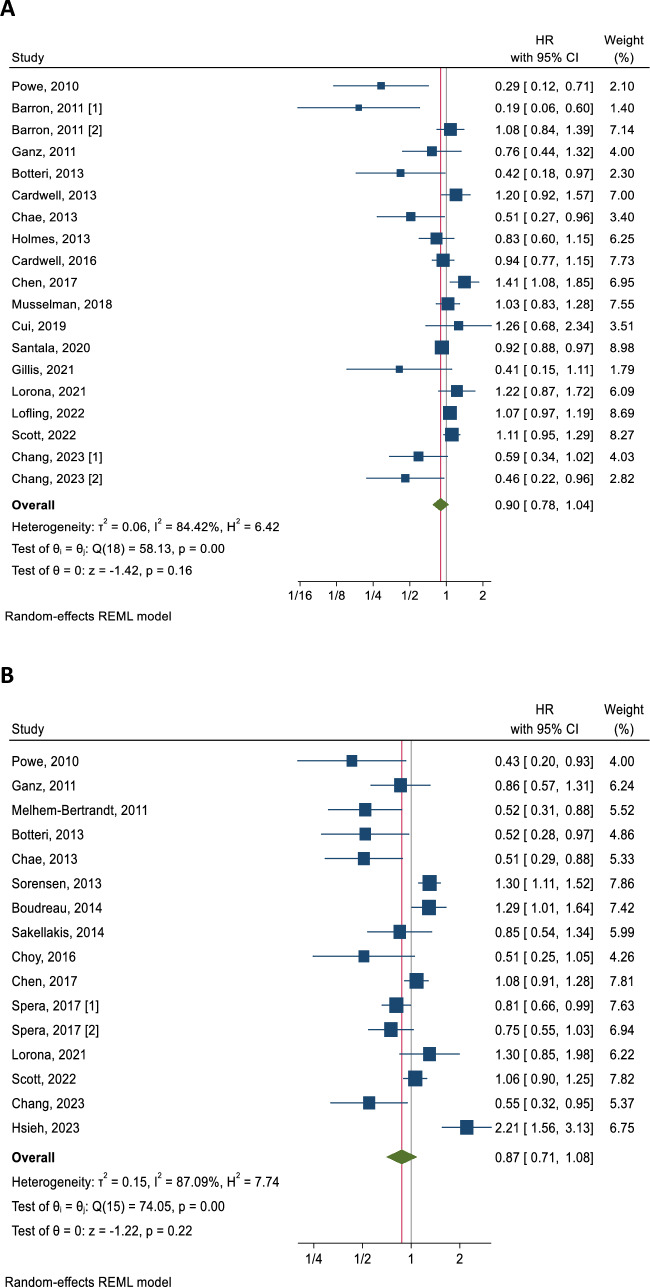
Forest plots for studies assessing the association between BB use and breast cancer prognosis. Legends (**a** breast cancer specific mortality, **b** breast cancer recurrence)

Overall, 17 studies (with two of these studies contributing two datasets each) were included in the meta-analysis examining the association between BB use and breast cancer specific death. Pooled results with a random effects model showed that there was no statistically significant association between BB use and BCD, with only a small reduction in the HR (HR = 0.90, 95% CI 0.78–1.04, *p* = 0.16; Fig. 2a). There was significant heterogeneity between these studies (*I*^2^ = 84.42%). Sensitivity analysis iteratively recalculating the HR by removing one study at a time did not significantly alter the results (HRs 0.86–0.96, all *p* > 0.05; Supplementary Fig. 1A). Further, the confidence intervals of all the meta-analyses while removing one study at a time included the HR of the overall summary estimate. Subgroup analyses showed that the presence of ITB may affect the outcome. In the studies judged to be susceptible to this bias, the results indicated a statistically significant protective association between BB use and BCD (HR = 0.42, 95% CI 0.25–0.71, *p* = 0.001, Table 2), but there was no association in the studies judged not to be susceptible (HR = 0.99, 95% CI 0.91–1.09, *p* = 0.89; *p* for subgroup difference = 0.002). Other analyses revealed that stratifying the association by other potential modifiers (exposure period and type of BB used) did not significantly change the results (*p*-values for subgroup differences both > 0.05).

**Table 2:** Subgroup meta-analyses

Study characteristics	Breast cancer specific mortality					Breast cancer recurrence				
	Number per subgroup	HR (95% CI)	*I*^2^ (%)	*P*_a_	*P*_b_	Number per subgroup	HR (95% CI)	*I*^2^ (%)	*P*_a_	*P*_b_
Immortal time bias										
No	17 [23, 36, 45–48, 50–54, 56–58, 64]	0.99 (0.91–1.09)	56	0.89		11 [23, 24, 36, 37, 46, 47, 52, 58, 60–62]	1.02 (0.80–1.29)	87	0.88	
Yes	2 [44, 49]	0.42 (0.25–0.71)	2	**0.001**	**0.002**	5 [44, 49, 59, 63]	0.67 (0.53–0.84)	39	**0.001**	**0.02**
Exposure periodc										
Prediagnosis	12 [23, 36, 45, 47, 48, 51, 55–58]	0.98 (0.93–1.04)	18	0.61		5 [23, 36, 37, 47, 58]	0.90 (0.66–1.22)	78	0.49	
Postdiagnosis	7 [48, 51–55, 58]	1.06 (0.94–1.20)	64	0.32	0.30	7 [24, 37, 52, 58, 60–62]	1.16 (0.91–1.49)	86	0.24	0.20
Type of BB_c_										
Selective	6 [45, 52, 53, 55, 57, 58]	1.03 (0.94–1.12)	45	0.55		4 [24, 37, 52, 58]	1.36 (0.91–2.03)	93	0.13	
;Nonselective	9 [45, 48, 51–53, 55–58]	1.04 (0.92–1.17)	28	0.54	0.94	4 [24, 37, 52, 58]	1.24 (1.01–1.53)	49	**0.04**	0.82

^a^*p*-values for specific subgroup hazard ratio

^b^*p*-values for subgroup differences. The differences were tested through a random-effects meta regression

^c^The ‘exposure period’ and ‘type of BB’ subgroups were restricted to studies judged not to be susceptible to ITB

^d^**Bold text** indicates a statistically significant *p*-value

### Association between BB use and breast cancer recurrence

Overall, 15 studies (with one of these studies contributing two datasets) were included in the meta-analysis examining the association between BB use and breast cancer recurrence. Pooled results with a random effects model showed that there was no statistically significant association between BB use and BCR, with only a small reduction in the HR (HR = 0.87, 95% CI 0.71–1.08, *p* = 0.22; Fig. 2b). There was significant heterogeneity between these studies (*I*^2^ = 87.09%). Sensitivity analysis iteratively recalculating the HR by removing one study at a time did not significantly alter the results (HRs 0.84–0.90, all *p* > 0.05; Supplementary Fig. 1B). Further, the confidence intervals of all the meta-analyses while removing one study at a time included the HR of the overall summary estimate. Subgroup analyses showed that the presence of immortal time bias may affect the outcome. In the studies judged to be susceptible to this bias, the results indicated a statistically significant protective association between BB use and BCR (HR = 0.67, 95% CI 0.53–0.84, *p* = 0.001, Table 2), but there was no association in the studies judged not to be susceptible (HR = 1.02, 95% CI 0.80–1.29, *p* = 0.88; p for subgroup difference = 0.02). Other analyses revealed that stratifying the association by other potential modifiers (exposure period and type of BB used) did not significantly change the results (p-values for subgroup differences both > 0.05).

### Publication bias

**Fig. 3:**
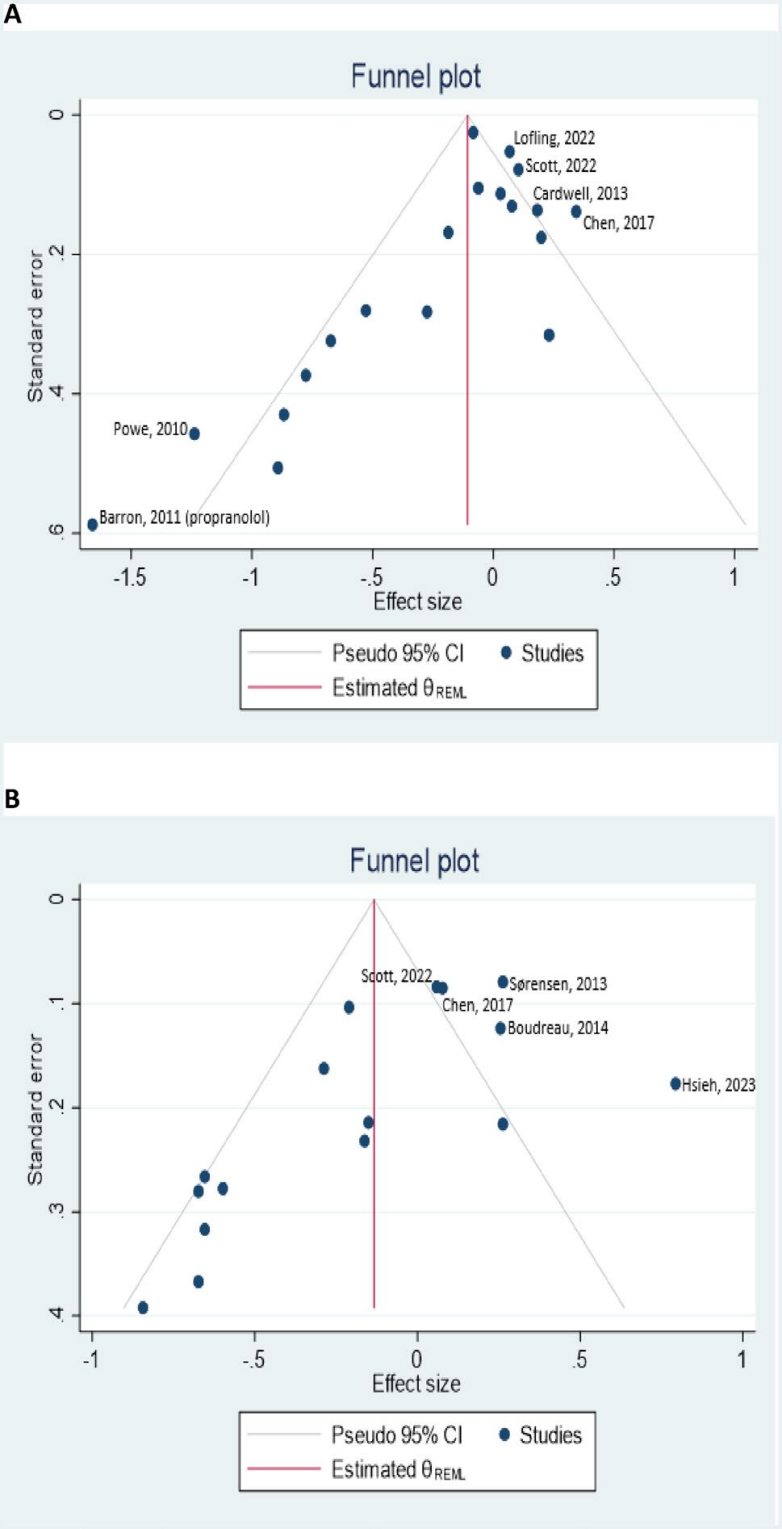
Funnel plots for studies assessing the association between BB use and breast cancer prognosis. The X axis represents HRs or ORs on the log scale. Legends (**a** breast cancer specific mortality, **b** breast cancer recurrence)

Funnel plots for the association between BB use and both BCD and BCR showed clear evidence of asymmetry and publication bias (Figs. 3a and 3b). In the plot with BCR as the outcome, there was asymmetry in terms of the number of studies on either side of the average effect size, with the plot having more studies on the left-hand side (meaning that there were more studies that showed a lower HR than the overall average effect size relative to studies that showed a higher HR). Further, there was very clear evidence of small study effects in both plots, with big clusters of small studies with very large effect sizes in the bottom left of the plots. The results of Egger’s regression tests for these outcomes were in concordance with a visual inspection of the respective funnel plots (p-values for small study effects < 0.05 in both plots).

## Discussion

In this meta-analysis, we found no statistically significant association between BB use and both BCD and BCR, with only a small reduction in the HR for both outcomes. The results were similar when leaving one study out at a time for each outcome, suggesting that the results are relatively stable and were not affected by the presence of outliers. These findings are consistent with some of the contemporary meta-analyses examining the association between BB use and breast cancer prognosis [18–22], which all found a small reduction in the HR for both BCD and BCR, although none of these associations reached statistical significance either. One recently published meta-analysis also found no association between BB use and BCD, but a small and statistically significant increased risk for BCR [65]. Two older meta-analyses [16, 17] both found a statistically significant protective association for BCD and a non-statistically significant risk reduction for BCR, however these meta-analyses included far fewer studies than both the current and more recently published meta-analyses. Furthermore, older meta-analyses were more influenced by older observational studies that were often prone to methodological limitations such as immortal time bias [25]. For example, the summary estimate for BCD in the meta-analysis by Childers and others [16] only included four studies, one of which was judged to be susceptible to ITB [44] in the current meta-analysis. The summary estimate for BCD in the meta-analysis by Raimondi and others [17] included the same studies as the meta-analysis by Childers et al., plus two additional studies, one of which was judged to be susceptible to ITB [49] in the current meta-analysis.

Immortal time bias (ITB) is a bias introduced in pharmacoepidemiological studies in which a survival advantage is conferred to the user group by way of misattributing user time over a period where events could not occur by design [25]. For example, if a postdiagnostic exposure period is used and a binary yes/no indicator is used to model medication use (i.e., a time fixed approach), the time between cancer diagnosis and the first medication dispensing is ‘immortal’ because the patient needed to have survived this period to be dispensed a medication. Lévesque and colleagues showed that medications with no biological basis for doing so (e.g., NSAIDS) could be made to show a decreased risk of diabetes progression by modelling medication use through a time fixed approach [66]. The subgroup analysis in this meta-analysis showed a similar (and expected) pattern, with statistically significant subgroup differences between studies judged to be susceptible to ITB relative to studies judged not to be susceptible to ITB for both outcomes. In fact, there was no longer a suggestion of a protective association for either outcome when studies judged to be susceptible to ITB were excluded from the analysis. Only one previous meta-analysis in this area has examined ITB as a potential effect modifier [19], and it found no suggestion of effect modification. The pooled HR with BCR as the outcome in this meta-analysis was 0.88 (0.66–1.17), and 0.83 (0.54–1.30) when removing studies judged to be susceptible to ITB. There were some disagreements between this meta-analysis and the current meta-analysis in terms of judging studies to be prone to be ITB or not. In the current meta-analysis, the studies by Boudreau et al. [60] and Ganz et al. [46] were judged not to be susceptible to ITB, while the study by Powe et al. [44] was. However, the other meta-analysis [19] judged the studies by Boudreau et al. and Ganz et al. to be prone to ITB, and the study by Powe et al. to not be prone to ITB. In the current meta-analysis, we made a strong effort to correctly classify studies in terms of their risk of ITB, including emailing authors to clarify their BB exposure definition.

There have been hypotheses that BBs used prior to diagnosis (or around the time of diagnosis) may be more efficacious in inhibiting breast cancer progression than BBs used after diagnosis, as this time is generally very stressful for the patient and BBs can aid in alleviating this stress [15, 27–33]. Furthermore, there may be a synergistic and beneficial interaction between BBs and chemotherapy, as stress is conjectured to inhibit the effectiveness of chemotherapy through β-adrenergic signalling [71–73]. Post curative surgery, however, the antimetastatic effects of BBs may be minimal relative to the time period around diagnosis. Despite a number of studies in each subgroup for both outcomes, our meta-analysis was unable to ascertain a difference between the two exposure periods. One recently published meta-analysis [20] found a suggestion of a more protective association for pre diagnosis use with BCR as the outcome (*p* for subgroup difference = 0.09), however the number of studies in each subgroup was small relative to the current meta-analysis. Furthermore, this meta-analysis did not exclude studies judged to be susceptible to ITB in their subgroup analyses.

In the subgroup analysis by type of BB, there was no specificity shown in terms of either type (selective or non-selective) being more efficacious than the other. Several preclinical studies have suggested that non-selective BBs may have a higher efficacy in inhibiting pathways involved in breast cancer progression and metastasis [15, 26]. In particular, propranolol has been shown to have antimigratory and antiangiogenic properties in both animal models and human cancer cell lines [67–71]. Despite this, the subgroup results by type were not suggestive of a differential effect, with nonsignificant subgroup differences found for both outcomes. A somewhat recently published meta-analysis also found little evidence of effect modification by type [21], however it is worthy to note that the number of studies included in this subgroup analysis were small for this meta-analysis as well the current meta-analysis. In the current meta-analysis, there were only four studies in each group for the recurrence outcome, which likely contributed to relatively unstable estimates.

It must be acknowledged that there was a significant amount of between study heterogeneity present in this meta-analysis, even in many of the individual arms of the subgroup analyses. As shown in Table 1, different studies were often conducted over different time periods and in different countries, inevitably meaning that BBs were often used in different clinical contexts. BBs were commonly indicated for hypertension 15–20 years ago, however heart failure is now their most common indication [9, 74]. As such, BB users are likely to be less healthy in contemporary studies relative to older studies. There was also little uniformity regarding the characteristics of women enrolled or analysed, with a range of different cancer stages and ages studied. Similarly, the covariates adjusted for varied widely across studies, with some studies making comprehensive adjustments for a range of covariates, while other studies likely lacked access to a similar range of potential confounders. Finally, even though the prediagnosis and postdiagnosis exposure periods were examined in subgroup analyses, there was also heterogeneity present within these groups. For example, postdiagnosis BB use may mean use in the first year after diagnosis only or use any time after diagnosis. All of these factors likely play a role in influencing any effect estimate derived, especially when > 1 of these factors differ from study to study.

We found clear evidence of publication bias and small study effects, with evidence of asymmetry in the funnel plot with BCR as the outcome and big clusters of small studies with very large effect sizes for both outcomes. In the main summary estimates, there was only a suggestion of a protective association for BBs, with non-statistically significant results for both outcomes. If publication bias was not present in this meta-analysis and the most imprecise studies with very large and protective effect sizes were omitted, it would mean that the overall HR would attenuate towards the null (1.0) for both outcomes. This would mean that we would have even less confidence in a potential protective effect for BBs, and along with the ITB subgroup analysis described above, gives credence to the hypothesis that BBs have little or no effect on the prognosis of breast cancer.

The main strength of this meta-analysis is the large number of observational studies included and the resulting total number of women with breast cancer analysed (253,082). Furthermore, we studied both BCD and BCR as outcomes, and the consistency in the results between these outcomes gives us confidence in the interpretation of the meta-analysis. We also conducted subgroup analyses by ITB, exposure period, and the type of BB used, which were all subgroups we were interested in a priori with legitimate methodological or clinical justifications for analysing.

This study is not without its limitations. As mentioned above, there was significant between study heterogeneity in this meta-analysis, and as a result, it is difficult to determine if the pooled effect estimates derived are driven by BB use exclusively, extraneous factors such as the study period and/or other modifying factors between studies, or a combination of these. Furthermore, even though preclinical evidence suggests that the effect of BBs may vary by subtype (and might be efficacious for triple negative breast cancer in particular) [33, 75, 76], we did not conduct a subgroup analysis by breast cancer type due to how rarely this was reported. Finally, although there have been suggestions that long term BB use may be beneficial in preventing the growth and/or spread of breast cancer [45, 58], we were unable to conduct a subgroup analysis by dose of BB due to how rarely this was reported as well.

In conclusion, we found no evidence of BBs being protective or increasing risk for the outcomes of BCD and BCR in this meta-analysis. Protective effects reported in some previous meta-analyses are likely due to studies with immortal time bias, and also to publication bias. We found no indication of effect modification by either the type of BB used (selective vs non-selective) or the exposure period in which BBs were used (prediagnosis vs postdiagnosis). However, our findings are based on observational studies and should be confirmed in future clinical trials. Such work should ideally focus on subgroups for which preclinical evidence of potential benefit has been found, such as specific types of BBs (e.g., non-selective) or subtypes of breast cancer (e.g., triple negative).

### Supplementary Information

Below is the link to the electronic supplementary material.

Supplementary file1 (DOCX 31 kb)

## Data Availability

Not applicable.
