# Supplementary material for: Beta-blocker use and breast cancer outcomes: a meta-analysis
Source: Breast Cancer Res Treat. 2024 Jun 5;206(3):443–63. doi: 10.1007/s10549-024-07263-4 (PMC11208256; doi:10.1007/s10549-024-07263-4)
Supplement: Supplementary file 1 — Supplementary file1 (DOCX 31 kb) [file 10549_2024_7263_MOESM1_ESM.docx]

**A**

**B**

Figure 1. Leave one out forest plots for studies assessing the association between BB use and breast cancer prognosis. Legends (**A**: breast cancer specific mortality; **B**: breast cancer recurrence)
